# Association of Obesity with Hearing Impairment in Adolescents

**DOI:** 10.1038/s41598-018-37739-5

**Published:** 2019-02-12

**Authors:** Franco Scinicariello, Yulia Carroll, John Eichwald, John Decker, Patrick N. Breysse

**Affiliations:** 1 Division for Toxicology and Human Health Services, Agency for Toxic Substances and Disease Registry, Atlanta, USA; 2 Office of Science, National Center for Environment Health, Centers for Disease Control and Prevention, Atlanta, USA; 3 Office of the Director, National Center for Environment Health, Centers for Disease Control and Prevention, Atlanta, USA

## Abstract

Hearing loss (HL) is the third most common chronic physical condition in the United States. Obesity has become an increasingly important public health concern, as the prevalence in children, adolescents and adults has increased over the past few decades. The objectives of this study is to investigate whether obesity is associated with audiometric notches indicative of noise-induced hearing loss (NIHL), speech frequency hearing loss (SFHL), and high frequency hearing loss (HFHL) in adolescent participants of the National Health and Nutrition Examination Survey 2007–2010. The prevalence of overall audiometric notches in the adolescent population was 16.0% with higher prevalence in females than males. The prevalence of SFHL and HFHL were higher in males than females (SFHL, 7.3% vs. 5.4%, respectively; and HFHL 14.3% vs. 8.1%, respectively). Obese adolescents had a higher adjusted OR to have audiometric notches (OR = 1.93; 95% CI: 1.33–2.81) and HFHL (OR = 1.95; 95% CI: 1.19–3.21). Continued preventative efforts towards reducing obesity might also help to reduce the risk for HL and NIHL.

## Introduction

Hearing loss (HL) is the third most common chronic physical condition in the United States^1^. The most common preventable cause of HL is the damage to the auditory system caused by excessive noise exposure^2,3^. Although the onset of this type of HL is usually slow, it progresses as long as the exposure continues. One of the first signs of noise-induced hearing loss (NIHL) is an audiometric indication of a noise-induced hearing threshold shift (NITS), usually defined as an “audiometric notch” at 3, 4, or 6 kHz. Henderson and colleagues^4^ reported increased prevalence of audiometric notches in adolescents: 15.9% in NHANES 1998–1994 to 16.8% in NHANES 2005–2006. Furthermore, damage to hearing at a young age can lead to exacerbated hearing loss later in life^2^. Data from the National Health and Nutrition Examination Survey (NHANES) suggest that the prevalence of HL among United States adolescents (12–19 years old**)** increased from 1994 to 2006 for both high frequency HL (HFHL) and low frequency HL (LFHL). HFHL increased from 11.1% to 12.9% and LFHL increased from 5.2% to 6.5%^4^.

Obesity has become an increasingly important public health concern, as the prevalence in children, adolescents and adults has increased over the past few decades both within the United States and worldwide. Among U.S. adolescents aged 12 to 19 years, obesity prevalence increased between 1988–1994 and 2013–2014, from 10.5% (95%CI: 8.8, 12.5) to 20.6% (95%CI: 16.2, 25.6)^5^.

Health problems during childhood and adulthood associated with obesity include an increased risk of diabetes, hypertension, nonalcoholic fatty liver disease, and depression^6^. Moreover, obesity has been associated with unilateral low frequency HL, but not unilateral high frequency HL, in adolescent participants to NHANES 2005–2006^7^. There are no studies about potential risk factors for audiometric notches in adolescents. Therefore, the objective of this study is to investigate whether obesity is associated with audiometric notches indicative of NIHL and hearing loss in adolescents in a nationally representative survey.

## Methods

### Study population

NHANES is a cross-sectional, nationally representative survey of the non-institutionalized civilian population of the United States conducted by the National Center for Health Statistics at the Centers for Disease Control and Prevention (CDC)^8^. Beginning in 1999, the survey has been conducted continuously and released in 2-year cycles. For our study, we merged the publicly available files for NHANES cycles 2007–2008 and 2009–2010 using the NCHS recommendations^8^. The survey employed a multistage stratified probability sample based on selected counties, blocks, households, and persons within households. During NHANES 2007–2010, 2,520 participants aged 12–19 years had complete audiometric data (response rate 97.8%, among 2577 participants who completed household interviews). We excluded participants with: 1) only a partial audio exam (missing information in any air conduction threshold, n = 147); 2) otoscopic screening exam of the ear canals and eardrum for excessive or impacted ear cerumen (wax), physical abnormalities, or collapsing external ear canals that was not normal (n = 475), or ear compliance ≤0.2 mL (N = 42), or pressure lower than −150 dekapascals (daPa) (n = 18), and; 3) tinnitus based on the positive answer to the question “In the past 12 months, have you been bothered by ringing, roaring or buzzing in your ears or head that lasted for five minutes or more?” (n = 119). We, also, excluded participants that had missed information in the co-variates used in the analyses (n = 250). Therefore, the total sample size used in our analyses was of 1469 adolescent participants.

### Audiometric Measurements

Trained examiners obtained audiometric measurements in a specifically designed and equipped Mobile Exam Center (MEC) sound-treated room using a standardized protocol.

Air conduction thresholds were measured for each ear at 0.5, 1, 2, 3, 4, 6, and 8 kHz. The 1-kHz frequency was tested twice in each ear to measure the reliability of the participant’s responses and the average test response at 1-kHz frequency was used in the analyses. Pure-tone audiograms were not accepted if the difference between the 1-kHz test-retest thresholds was 10 dB or greater. For further details about the measurement techniques, see the National Center for Health Statistics website

[https://wwwn.cdc.gov/nchs/nhanes/continuousnhanes/manuals.aspx?BeginYear=2007; https://wwwn.cdc.gov/nchs/nhanes/continuousnhanes/manuals.aspx?BeginYear=2009].

### Definition of Audiometric Notch and Hearing Loss

In this study, we define the presence of a high-frequency audiometric notch as reported by Carroll *et al*.^9^ when:

- one or more of the thresholds (the softest sound a person can hear) at 3, 4, or 6 kHz exceeds the pure-tone average (PTA) of the 0.5 and 1 kHz thresholds by 15 dB or more, and
- the 8 kHz threshold is at least 5 dB lower (better) than the maximum threshold in the 3, 4, or 6 kHz range.

Similar to other studies using NHANES, we used the average of four audiometric frequencies at 0.5, 1, 2, and 4 kHz to define the speech frequency (SF) PTA^10,11^ and the average of the three audiometric frequencies at 3, 4, and 6 kHz to define an high frequency (HF) PTA^4,10,11^. We used a PTA of 15 dB HL or greater in either ear as a cutoff threshold for both the SF and HF PTA to define speech frequency hearing loss (SFHL) and high frequency hearing loss (HFHL), respectively^4,7,11^.

### Sociodemographic and hearing-related variables

In our analyses, we used sociodemographic and hearing-related variables that have or are suspected to have an effect on hearing thresholds. These variables used for our analysis were age, sex, race/ethnicity, poverty income ratio (PIR), body weight status (normal, underweight, overweight, and obese), serum cotinine, self-reported ear infection, and “exposure outside of a job to steady loud noise or music for 5 or more hours a week.”

We categorized race/ethnicity as non-Hispanic white, non-Hispanic black, Mexican American, and other (other Hispanic and other race). PIR is a measure of socioeconomic status and represents the calculated ratio of household income to the poverty threshold after accounting for inflation and family size.

Body mass index (BMI) is calculated by dividing the weight by height squared (kg/m^2^). However, since the relation between BMI and body weight in children depends on age and sex, obesity was defined as a BMI at or above the 95th percentile of the CDC sex-specific BMI-for-age growth charts from 2000^12^. Overweight was defined as a BMI between the 85th and 95th percentiles. Underweight was defined as BMI less than the 5^th^ percentile. Normal weight was defined as BMI between the 5^th^ percentile to less than the 85^th^ percentile.

Serum cotinine has been used as a biomarker of exposure to both environmental and/or active tobacco smoke. In addition to serum cotinine, NHANES questionnaire on “recent tobacco use” report information about the self-report use of tobacco in past 5 days. For youth 12–19 years of age, the questions on tobacco use were self-administered using the Audio Computer-Assisted Self-Interview system. Therefore, tobacco exposure was categorized in: 1) no smokers, consisting of participants that did not report use of tobacco in the past five days and had serum cotinine below the limit of detection (LOD = 0.015 ng/ml); 2) second hand smokers exposure (SHS), defined as person who did not use tobacco in the past 5 days and had serum cotinine at or higher than the LOD and below 10 ng/ml^13^; 3) smokers, defined as participants who reported use of tobacco in the past 5 days or serum cotinine level ≥10 ng/mL.

The household interview gathered information about self-reported ear infection and “exposure outside of a job to steady loud noise or music for 5 or more hours a week.” Generally, persons 16 years of age and older and emancipated minors were interviewed directly, whereas a responsible adult provided information for participants under 16 years of age.

The NHANES physical activity questionnaire reports information on time of sedentary activity: “How much time do you usually spend sitting on a typical day?” The participants were asked ‘about sitting at work, at home, getting to and from places, or with friends, including time spent sitting at a desk, traveling in a car or bus, reading, playing cards, watching television, or using a computer. Excluding the time spent sleeping, how much time do you usually spend sitting on a typical day?’ Since the variable was right skewed, it was naturally log-transformed.

### Statistical Methods

All analyses were performed using the Mobile Exam Center weight as recommended by NCHS, and to account for the complex sampling design and non-response of NHANES^8^. SAS 9.3 (SAS Institute, Cary, NC) and SAS-Callable SUDAAN 10 (Research Triangle Institute, Research Triangle Park, NC) was used for all statistical analyses. We used logistic regression to calculate adjusted odds ratios (ORs) and 95% CIs for the audiometric notch and the hearing loss outcomes. Preliminary analyses did not found any significant interaction term between the predictors (p > 0.10). Statistical tests for linear trends were conducted by modeling ordinal variable using integer values. Two models, utilizing the various covariates were run: (1) model 1 included age, sex, race/ethnicity, obesity, PIR, ear infections, loud noise exposure and smoking; (2) and, as sensitivity analyses, model 2 included the covariates in model 1 plus hours of sedentary activity.

## Results

Table 1 shows the characteristics of the population. Among adolescents, 63.9% were non-Hispanic white; 48.2% were female; 18.4% were obese, and 21.5% were from families with income at or below the poverty level. Thirty-six percent and 24.4% of adolescents reported three or more ear infections or exposure to loud noise for 5 or more hours per week, respectively. Second hand smokers were 56.8% and 16.0% were smokers. The weighted prevalence of audiometric notches, SFHL and HFHL in adolescents was 16.0%, 6.3%, and 11.3%, respectively.

**Table 1:** Weighted characteristics of adolescent participants (12–19 years of age) in NHANES 20072010.

	n = 1469
Any Audiometric Notch, % (SE)	16.0 (0.95)
Bilateral Audiometric Notch, % (SE)	1.5 (0.39)
Unilateral Audiometric Notch, % (SE)	14.5 (1.02)
Speech Frequency Hearing Loss, % (SE)	6.3 (0.98)
High Frequency Hearing Loss, % (SE)	11.3 (1.51)
Male, % (SE)	51.8 (1.83)
Female, % (SE)	48.2 (1.83)
**Age**	
12–15 years, % (SE)	51.1 (1.35)
18–19 years, % (SE)	48.9 (1.35)
**Race/Ethnicity**	
Non-Hispanic White, % (SE)	63.9 (2.92)
Non-Hispanic Black, % (SE)	12.2 (1.10)
Mexican-American, % (SE)	11.8 (1.66)
Other (Other Hispanic and Other race), % (SE)	12.1 (1.77)
**Poverty Income ratio**	
PIR ≤ 1, % (SE)	21.5 (1.62)
PIR > 1, % (SE)	78.5 (1.62)
**Body Weight Status**	
Obese, % (SE)	18.4 (1.25)
Overweight, % (SE)	16.0 (1.06)
Underweight, % (SE)	3.3 (0.58)
Normal, % (SE)	62.3 (1.21)
Ever had 3 or more ear infections, % (SE)	36.0 (1.98)
Loud noise exposure for 5 or more hours per week, % (SE)	24.4 (1.44)
**Smoking status**	
no smokers, % (SE)	27.2 (1.93)
second hand smokers exposure (SHS), % (SE)	56.8 (1.1.67)
smokers, % (SE)	16.0 (1.12)
Serum Cotinine ng/mL; GM (SE)	0.2 (0.02)
Hours of sedentary Activity; GM (SE)	6.2 (0.16)

### Audiometric Notches

Table 2 shows the prevalence of overall audiometric notches. Females had a higher prevalence of overall notches than males (18.2% vs 13.9%, χ^2^ p value = 0.06). The weighted prevalence of audiometric notches in obese adolescents was higher compared to normal weight adolescents (24.8% vs 14.7%).

**Table 2:** Estimated population prevalence % (95% CI) of audiometric notches and hearing Loss in US adolescents aged 12 to 19 years, NHANES 2007–2010.

	Audiometric Notches		SFHL		HFHL	
	% (SE)	χ^2^ p value	% (SE)	χ^2^ p value	% (SE)	χ^2^ p value
Male, % (SE)	13.9 (1.10)		7.3 (1.68)		14.3 (2.43)	
Female, % (SE)	18.2 (1.76)	0.06	5.4 (0.93)	0.33	8.1 (1.21)	0.02
**Age**						
12–15 years, % (SE)	16.2 (1.49)		5.6 (1.12)		10.2 (1.33)	
18–19 years, % (SE)	15.8 (1.21)	0.82	7.1 (1.35)	0.32	12.4 (2.44)	0.39
**Race/Ethnicity**						
Non-Hispanic White, % (SE)	16.3 (1.49)		5.6 (1.42)		11.6 (2.29)	
Non-Hispanic Black, % (SE)	12.5 (1.45)		7.0 (1.35)		8.9 (1.84)	
Mexican-American, % (SE)	18.5 (1.97)	0.07	8.4 (1.54)	0.58	13.8 (1.40)	0.17
Other (Other Hispanic and Other race), % (SE)	15.4 (2.4)		7.4 (1.80)		9.6 (2.36)	
**Poverty Income ratio**						
PIR ≤ 1, % (SE)	16.7 (1.97)		6.1 (1.15)		12.2 (1.78)	
PIR > 1, % (SE)	15.8 (1.15)	0.69	6.4 (1.15)	0.83	11.0 (1.78)	0.60
**Body Weight Status**						
Obese, % (SE)	24.8 (2.30)		8.5 (1.81)		17.9 (3.22)	
Overweight, % (SE)	12.7 (2.16)		8.3 (2.51)		10.3 (2.14)	
Underweight, % (SE)	14.7 (7.99)	0.01	3.5 (2.62)	0.15	14.5 (7.14)	0.06
Normal, % (SE)	14.3 (1.37)		5.4 (1.02)		9.4 (1.60)	
Ever had 3 or more ear infections, % (SE) YES	16.7 (1.71)		8.7 (1.38)		12.0 (2.06)	
Ever had 3 or more ear infections, % (SE) NO	15.6 (1.25)	0.59	5.0 (1.05)	0.02	10.9 (1.52)	0.51
Loud noise exposure for 5 or more hours per week, % (SE) YES	19.5 (3.33)		7.6 (1.66)		16.4 (2.77)	
Loud noise exposure for 5 or more hours per week, % (SE) NO	14.8 (1.55)	0.30	5.9 (1.06)	0.35	9.6 (1.49)	0.02
No smokers	12.6 (1.84)		5.0 (1.22)		6.7 (1.38)	
Second hand smokers exposure (SHS),	17.9 (1.01)	0.07	5.3 (0.92)	0.19	10.8 (1.60)	0.01
Smokers	15.1 (3.20)		12.6 (4.75)		21.0 (5.30)	

In multivariate logistic regression analyses (Table 3), obese adolescents had higher odds to have audiometric notches as normal weight adolescents [adjusted OR (aOR) = 1.93; 95% CI: 1.33–2.81. p trend = 0.002). Further analyses with hours of sedentary activity (Table 3, Model 2), did not change the statistical significance association. As shown in Table 3, female adolescents had higher odds to have audiometric notches compared to males (aOR = 1.47; 95% CI: 1.06–2.03) and those exposed to SHS had higher odds to have audiometric notches compared to those not exposed to tobacco (aOR = 1.45; 95% CI: 1.04–2.00). Further analyses with hours of sedentary activity (did not change the statistical significance association (Table 3, Model 2).

**Table 3:** Multivariate logistic regression adjusted OR (95% CL) of having any audiometric notch for US adolescents aged 12 to 19 years, NHANES 2007–2010 using.

	Audiometric Notches	
	Model 1	Model 2
n. case/n. total	235/1469	233/1460
Male	Referent	Referent
Female	1.47 (1.06, 2.03)	1.51 (1.07, 2.11)
**Age**		
12–15 years	Referent	Referent
16–19 years	0.91 (0.66, 1.27)	0.88 (0.63, 1.23)
**Race/Ethnicity**		
Non-Hispanic White	Referent	Referent
Non-Hispanic Black	0.70 (0.51, 0.97)	0.69 (0.49, 0.96)
Mexican-American	1.22 (0.81, 1.84)	1.23 (0.83, 1.84)
Other (Other Hispanic and Other race)	0.95 (0.59, 1.51)	0.95 (0.60, 1.52)
**Poverty Income ratio**		
PIR ≤ 1	1.01 (0.67, 1.51)	0.99 (0.66, 1.47)
PIR > 1	Referent	Referent
Obese	1.93 (1.33, 2.81)	1.94 (1.34, 2.82)
Overweight	0.84 (0.52, 1.34)	0.83 (0.51, 1.35)
Underweight	1.12 (0.28, 4.52)	1.11 (0.27, 4.58)
Normal	Referent	Referent
No smokers	Referent	Referent
Second hand smokers exposure (SHS),	1.45 (1.04, 2.00)	1.44 (1.04, 2.00)
Smokers	1.26 (0.69, 2.31)	1.28 (0.69, 2.37)
**Ever had 3 or more ear infections?**		
Yes	1.05 (0.75, 1.47)	1.06 (0.75, 1.49)
No	Referent	Referent
**Loud noise exposure for 5 or more hours per week?**		
Yes	1.42 (0.76, 2.65)	1.43 (0.77, 2.68)
No	Referent	Referent
Hours sedentary activity(natural log-transformed)	—	0.92 (0.74, 1.16)

### Speech Frequency Hearing Loss (SFHL)

The prevalence of SFHL was 7.3% in males and 5.4% in females (Table 2). The weighted prevalence of SFHL in obese adolescents was higher compared to normal weight adolescents (8.5% vs 5.4%), but the difference was not statistically significant. In multivariate analysis no statistically significant association was found between SFHL and body weight status (Table 4).

**Table 4:** Adjusted OR (95% CL) from multivariate logistic regression of having hearing loss for US adolescents aged 12 to 19 years, NHANES 2007–2010.

	Speech Frequency Hearing Loss		High Frequency Hearing Loss	
	Model 1	Model 2	Model 1	Model 2
n. case/n. total	100/1469	99/1460	165/1469	165/1460
Male	Referent	Referent	Referent	Referent
Female	0.85 (0.46, 1.57)	0.85 (0.46, 1.57)	0.63 (0.39, 1.02)	0.63 (0.39, 1.03)
**Age**				
12–15 years	Referent	Referent	Referent	Referent
16–19 years	1.10 (0.62, 1.96)	1.13 (0.60, 2.12)	0.93 (0.54, 1.61)	0.92 (0.53, 1.60)
**Race/Ethnicity**				
Non-Hispanic White	Referent	Referent	Referent	Referent
Non-Hispanic Black	1.75 (0.89, 3.45)	1.80 (0.89, 3.62)	0.80 (0.43, 1.51)	0.79 (0.43, 1.48)
Mexican-American	2.28 (1.20, 4.31)	2.30 (1.18, 4.46)	1.43 (0.92, 2.24)	1.42 (0.91, 2.21)
Other (Other Hispanic and Other race)	1.77 (0.75, 4.19)	1.79 (0.76, 4.26)	0.85 (0.39, 1.86)	0.85 (0.39, 1.84)
**Poverty Income ratio**				
PIR ≤ 1	0.77 (0.45, 1.32)	0.79 (0.46, 1.35)	0.98 (0.59, 1.62)	1.10 (0.74, 1.64)
PIR > 1	Referent	Referent	Referent	Referent
Obese	1.52 (0.86, 2.70)	1.55 (0.87, 2.74)	1.95 (1.19, 3.21)	1.94 (1.18, 3.17)
Overweight	1.43 (0.71, 2.89)	1.48 (0.73, 2.98)	0.99 (0.59, 1.67)	1.00 (0.59, 1.70)
Underweight	0.51 (0.08, 3.09)	0.52 (0.08, 3.20)	1.33 (0.40, 4.43)	1.3. (0.39, 4.39)
Normal	Referent	Referent	Referent	Referent
No smokers	Referent	Referent	Referent	Referent
Second hand smokers exposure (SHS),	1.03 (0.50, 2.12)	1.00 (0.50, 2.03)	1.55 (0.78, 3.10)	1.56 (0.78, 3.12)
Smokers	2.82 (0.98, 8.08)	2.77 (0.99, 7.79)	3.06 (1.14, 8.21)	3.07 (1.17, 8.09)
**Ever had 3 or more ear infections?**				
Yes	2.12 (1.19, 3.76)	2.07 (1.17, 3.68)	1.09 (0.73, 1.64)	1.10 (0.74, 1.64)
No	Referent	Referent	Referent	Referent
**Loud noise exposure for 5 or more hours per week?**				
Yes	1.05 (0.63, 1.78)	1.07 (0.63, 1.82)	1.48 (0.95, 2.32)	1.48 (0.95, 2.30)
No	Referent	Referent	Referent	Referent
Hours sedentary activity(natural log-transformed)	_	1.02 (0.61, 1.72)	_	0.96 (0.68, 1.37)

### High Frequency Hearing Loss

The prevalence of HFHL (Table 2) was 14.3% among males and 8.1% among female adolescents, and the difference was statistically significant (χ^2^ p value = 0.02). The weighted prevalence of HFHL in obese adolescents was statistically significant higher compared to normal weight adolescents (17.9% vs 5.4%), (Table 2). In multivariate analyses (Table 4), obese adolescents had higher odds to have HFHL compared to the normal weight adolescents (aOR = 1.95; 95% CI: 1.19–3.21. p trend < 0.05) (Table 4, Model 1). The association remained when the hours of sedentary activity was, further, added as co-variate in the model (Table 4, Model 2). Furthermore, the odds of having HFHL were higher in smokers (aOR = 3.07; 95% CI: 1.14–8.21) compared to non-smokers (Table 4, Model 1).

## Discussion

In the present study conducted using data from the NHANES 2007–2010 surveys we found that obesity was associated with increase odd of having audiometric notches and high frequency hearing loss, but not with speech frequency HL. These findings are notable because of the potential impact that hearing impairment at high frequency can have on the social behavior of children and adolescents. Children with HL are more likely to experience academic difficulties, have behavioral problems, and demonstrate lower performance in oral language, compared with their peers with normal hearing^14^. Although the primary objective in this study was the potential association of obesity with hearing impairment, we found that tobacco exposure was, also, associated with audiometric notches and HFHL. In a previous analyses of adolescent participants of the NHANES 2005–2006, Lalwani *et al*.^7,15^ found that obesity^7^ and serum cotinine^15^ were associated with low frequency HL, but not unilateral high frequency HL. Although, there is a scarcity of epidemiological studies on the association of hearing impairment with body weight status in adolescents, several studies have reported an association of obesity with hearing loss in adults. In a longitudinal population-based cohort study (1993–95 to 2009–10), Cruickshanks *et al*.^16^ followed up 1925 participants (mean age 60.7 years at baseline) with a normal hearing baseline. They found that smoking and obesity were predicted risk factors for hearing loss (defined as a hearing threshold >25 dB of the pure tone average of 0.5, 1, 2, and 4 kHz in either ear). In a cross-sectional study comprising 61,052 Korean adults 30 years and older (20.13% female), Kim *et al*.^17^ reported that severely obese (BMI ≥ 30 kg/m^2^) persons had higher OR of hearing loss. Obesity was slightly associated with HL. Üçler *et al*.^18^ reported an association between obesity and hearing threshold in women aged 18–40 years. The associations were statistically significant at high frequencies of 4, 6, and 8 kHz, but not at lower frequencies.

In a prospective study conducted among 68421 women participants in the Nurses’ Health Study II from 1989 to 2009, Curham *et al*.^19^ reported that higher BMI and larger waist circumference were associated with increased risk of self-reported hearing loss. Conversely, Shargorodsky *et al*.^20^, in a study conducted in 26,917 adult men participants in the Health Professionals Follow-up Study, did not find any association between obesity with increased risk of self-reported hearing loss, but past smoking was independently associated with increased risk of hearing loss.

The biological plausibility of a role of obesity as risk factor for hearing impairment maybe through the perturbation of the adiponectin hormone. Hwang *et al*.^21^ hypothesized that plasma adiponectin mediates the effect of obesity on hearing loss. Adiponectin is a protein hormone that modulates several metabolic processes, among them glucose regulation and fatty acid oxidation^22^. Serum levels of adiponectin decrease with obesity^23^.

However, the best evidence supporting a role of obesity with the development of hearing impairment comes from *in vivo* studies Tanigawa *et al*.^24^ found that compared to the wild-type (WT) mice, adiponectin-knockout (APN-KO) mice had exacerbated hearing impairment, particularly in the high frequency range, with reduced cochlear blood flow and capillary density of the *stria vascularis*. The hearing impairment was prevented in APN-KO mice that were supplemented with adiponectin. Compared to the control group, Hwang *et al*.^25^ found that the auditory brainstem response threshold was significantly higher at high frequencies in mice with diet-induced obesity (DIO). Although susceptibility to damage from noise exposure is highly variable in the general population^26^, the potential reduction of cochlear blood flow in obese adolescents might make them more susceptible to the harmful effects of noise.

Our study has several limitations. The cross-sectional nature of this study limits the inferences that can be made based on the results. The associations reported in this study could be biased by uncontrolled factors such as genetic predisposition, otosclerosis, hypertension, diabetes, ototoxic medication, and exposure to ototoxic substances^27^; however, we adjusted the models for several important confounding factors. A major study limitation is the measurement of audiometric thresholds at a single point in time, since the identified audiometric notches may only represent temporary threshold shifts. Concerns have been raised about potential false-positive rates of high frequency audiogram notches, such as 6 and 8 kHz, which might be due to high subject variability and calibration error for TDH-type headphones^28^.

## Conclusion

Being obese was associated with NIHL and hearing loss. Health care professionals should be aware of the increased risk of damage to hearing among their patients with obesity. Since hearing impairment often progresses insidiously for years before being self-perceived or diagnosed^29^, early basic hearing screenings such as whisper or finger rub tests may provide early diagnosis and opportunities for noise prevention counselling and access to hearing aids^30^. Continued preventative efforts towards reducing obesity, should also have a positive impact among adolescents at risk for NIHL and hearing loss.
